# Supplementary material for: A Multifactorial Approach to Untangle Graphene Oxide (GO) Nanosheets Effects on Plants: Plant Growth-Promoting Bacteria Inoculation, Bacterial Survival, and Drought
Source: Nanomaterials (Basel). 2021 Mar 18;11(3):771. doi: 10.3390/nano11030771 (PMC8002891; doi:10.3390/nano11030771)
Supplement: Supplementary file 1 [file nanomaterials-11-00771-s001.pdf]

Supplementary Materials

A Multifactorial Approach to Untangle Graphene Oxide (GO) Nanosheets Effects on Plants: Plant Growth-Promoting Bacteria Inoculation, Bacterial Survival, and Drought

Tiago Lopes [1], Catarina Cruz [1], Paulo Cardoso [2], Ricardo Pinto [2], Paula A.A.P. Marques[3] and Etelvina Figueira [2,*]

[1] Department of Biology, University of Aveiro, Aveiro, Portugal; tslopes@ua.pt (T.L.); catarinasilvacruz@ua.pt (C.C.)

[2] Department of Biology & CESAM- Centre for Environmental and Marine Studies, University of Aveiro, Aveiro, Portugal; pjcardoso@ua.pt (P.C.); rl.pinto@ua.pt (R.P.)

[3] Department of Mechanics & TEMA- Centre for Mechanical Technology and Automation, University of Aveiro, Aveiro, Portugal; paulam@ua.pt

* Correspondence: efigueira@ua.pt

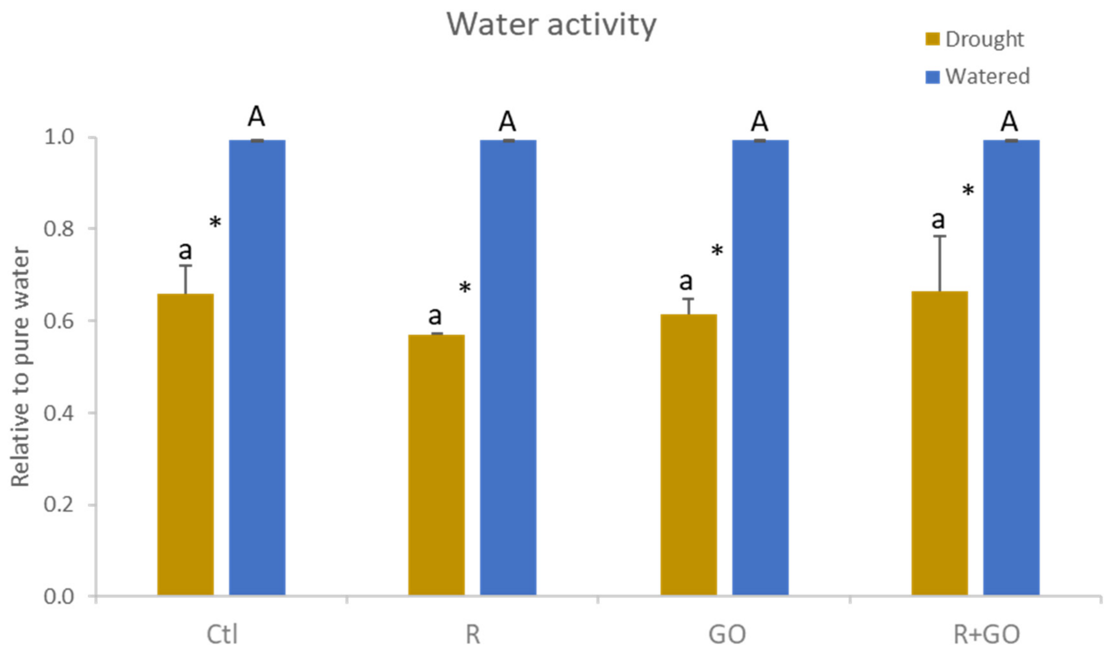

Figure S1. Water activity in the sand. Ctl- no addition of graphene oxide nanosheets, no bacterial inoculation; R- no addition of graphene oxide nanosheets, inoculation with *Rhizobium* strain E20-8; GO- addition of graphene oxide nanosheets, no bacterial inoculation; R+GO- addition of graphene oxide nanosheets, inoculation with *Rhizobium* strain E20-8.
